# Developmental Rhythm and Differential Accumulation of Flavonoids in Single- and Double-Flowered *Rosa xanthina*

**DOI:** 10.3390/plants15172669

**Published:** 2026-08-31

**Authors:** Na Li, Hairong Fan, Ruifen Ren, Zhihong Liu, Xiuyun Yang

**Affiliations:** 1 School of Architectural Engineering, Shanxi College of Applied Science and Technology, Taiyuan 030062, China; linared2004@163.com; 2 Shanxi Key Laboratory of Efficient Cultivation of Forest Resources, College of Forestry, Shanxi Agricultural University, Jinzhong 030801, China; 3 Key Laboratory of National Forestry and Grassland Administration on Forest Therapy and Wellness, College of Forestry, Shanxi Agricultural University, Jinzhong 030801, China

**Keywords:** petal type, metabolic difference, flowe-fruit development, active constituents, flavonoid accumulation

## Abstract

To clarify the utilization value of *Rosa xanthina* germplasm resources, single-flowered and double-flowered *Rosa xanthina* were used as experimental materials. The dynamic accumulation of total flavonoids and four flavonoid-related substances was determined in three floral developmental stages and three fruit developmental stages of single-flowered *Rosa xanthina.* Total flavonoids, rutin, hyperoside, quercetin and anthocyanins were measured by aluminum nitrate colorimetry, high-performance liquid chromatography (HPLC) and extraction method, respectively. Combined with correlation analysis and principal component analysis (PCA), the results showed statistically significant differences in flavonoid components during flowering stage (*p* < 0.05). The contents of total flavonoids and rutin in single-flowered *Rosa xanthina* were generally higher than those in double-flowered ones, while double-flowered *Rosa xanthina* had advantages in hyperoside and quercetin contents at the senescence stage. In fruit stage, flavonoid contents in single-flowered *Rosa xanthina* decreased significantly, and anthocyanins peaked at maturity. Correlation analysis revealed positive and negative statistical associations among flavonoid components, and PCA effectively distinguished samples from different developmental stages and component levels. This study clarified the component accumulation advantages of single-flowered and double-flowered *Rosa xanthina*, and screened key evaluation indexes, providing a scientific basis for differentiated utilization, precise harvesting and targeted regulation of *Rosa xanthina* varieties.

## 1. Introduction

Flavonoids are vital secondary metabolites in plants, which possess antioxidant, anti-inflammatory and antimicrobial activities, exhibiting prominent application potential in the development of functional foods and natural pharmaceuticals. Subclasses of flavonoids including anthocyanins and flavonols can scavenge intracellular reactive oxygen species and activate the Nrf2 antioxidant signaling pathway to alleviate oxidative damage. They exert health-promoting and clinical effects in cardiovascular and cerebrovascular protection as well as intervention against neurodegenerative diseases, serving as core raw materials for the exploitation of natural functional preparations [1,2]. Rutin, quercetin and hyperin are widely distributed in ornamental and medicinal plants belonging to Rosaceae and Tiliaceae. As a free flavonoid aglycone, quercetin presents powerful antioxidant capacity, while rutin and hyperin are its glycoside derivatives. The three compounds can jointly exert antioxidant, anti-inflammatory and organ-protective effects, and have become key research candidates for natural antioxidant additives and adjuvant raw materials for chronic disease management [2].

Numerous studies have demonstrated that flavonoid accumulation in plants exhibits remarkable tissue specificity and developmental dependence. The contents of total flavonoids and key monomeric compounds such as rutin and quercetin undergo regular dynamic variations along with plant growth and phenological shifts [1,2,3,4]. Floral investigations have revealed that flavonoid components in the petals of safflower and *Tilia miqueliana* display flower-stage-specific accumulation patterns. Monomers including quercetin and kaempferol reach maximum concentrations at specific flowering stages, with their abundances fluctuating dynamically as flowering proceeds [3,4]. In vegetative organs and fruits, flavonoid biosynthesis and accumulation in cotton leaves, *Rosa roxburghii* fruits, and *Astragalus membranaceus* are tightly regulated by developmental programs. Distinct plant species differ drastically in their dominant flavonoid constituents and accumulation rhythms, which highlights the sophisticated regulatory network governing plant flavonoid secondary metabolism [5,6,7]. Previous relevant studies merely focus on temporal flavonoid fluctuations within a single flower morphotype or isolated organ, and systematic comparative analyses of flavonoid monomer accumulation disparities during floral and fruit development across different flower variants of the same species remain scarce.

*Rosa xanthina* Lindl., a native deciduous shrub belonging to the genus Rosa of the Rosaceae family in northern China, boasts abundant natural resources and strong ecological adaptability. Among its variants, *Rosa xanthina* f. *normalis* is the wild original form, while *Rosa xanthina* ‘*Plena*’ is an artificially cultivated ornamental form, and the two differ markedly in floral morphology [8,9]. In terms of phenological traits, both variants bloom from April to June; only the single-flowered variant can bear fruit normally, whereas the double-flowered cultivar fails to complete fruit development due to stamen petalody [9,10]. *Rosa xanthina* possesses high ornamental value, and its flowers and fruits are rich in bioactive constituents including total flavonoids, rutin, hyperin and anthocyanins, which exhibit multiple pharmacological activities such as antioxidant, anti-inflammatory and lipid-regulating effects, showing great potential for economic exploitation and ecological utilization [11,12,13].

Previous studies have characterized flavonoid constituents of *Rosa xanthina* via chromatographic techniques including UPLC-Q-TOF-MS and HPLC, laying a foundation for research on bioactive compounds of this species [14,15,16]. Nevertheless, few studies have systematically compared dynamic flavonoid variations across distinct flower morphotypes throughout the entire developmental cycle, and continuous temporal data are lacking, which restricts the in-depth exploitation of its resources. Accordingly, this study employed single-flowered and double-flowered *Rosa xanthina* as experimental materials to quantify the contents of core flavonoids at different developmental stages of flowers and fruits. We aimed to clarify the regulatory patterns of cultivar type and organ tissue on flavonoid accumulation, identify the optimal developmental stages for maximum bioactive compound accumulation and the superior utilizable organs, fill the research gaps in secondary metabolism of *Rosa xanthina*, and provide theoretical references for germplasm breeding, targeted cultivation, and industrialized extraction of natural flavonoids.

## 2. Results

### 2.1. Variation Patterns of Flavonoids in Rosa xanthina f. normalis and Rosa xanthina ‘Plena’ at Different Stages

**Figure 1:**
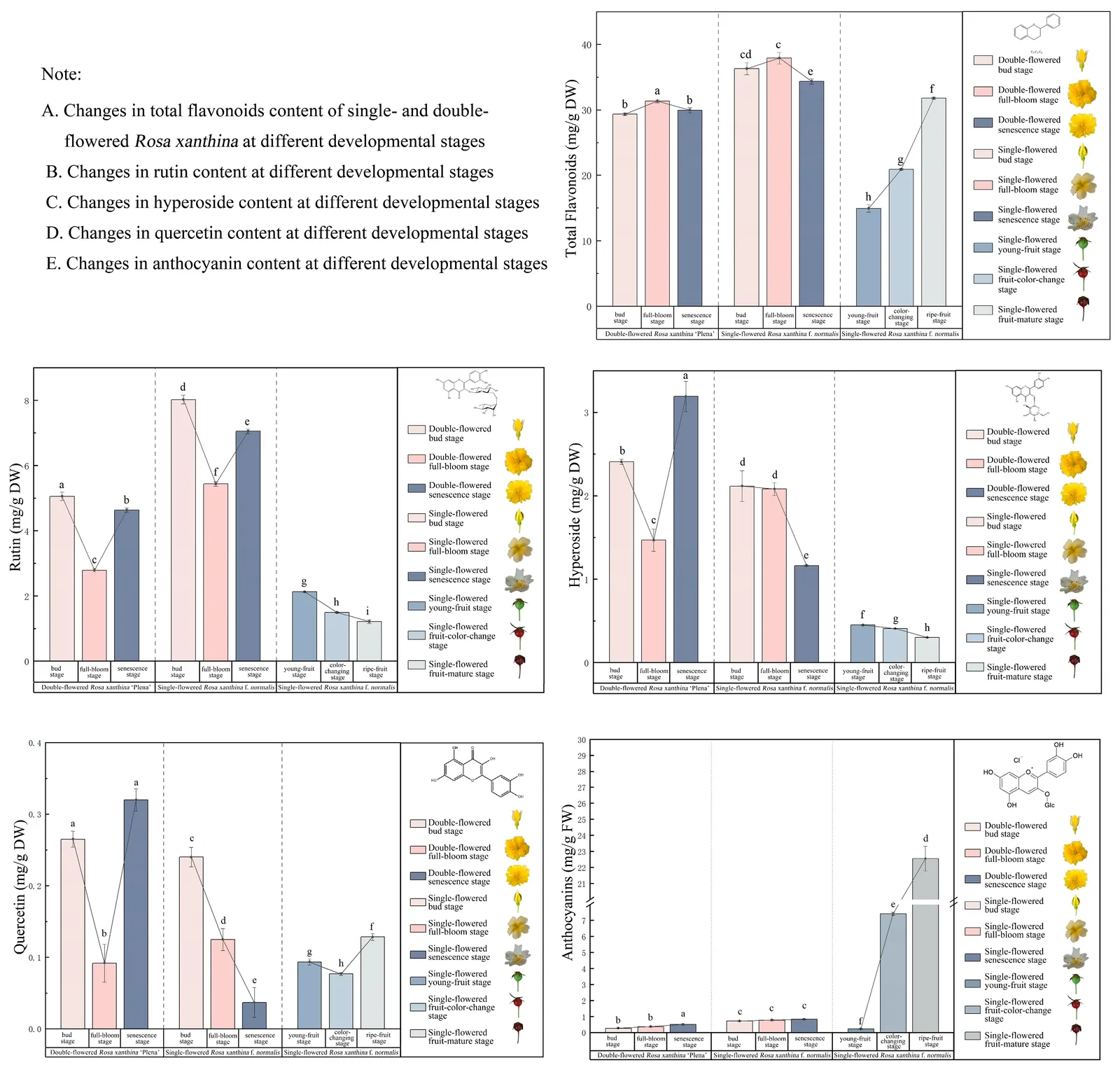
Changes in total flavonoids and four flavonoid-related substances of single- and double-flowered *Rosa xanthina* at different developmental stages. Different lowercase letters above bars indicate significant differences according to one-way ANOVA at *p* < 0.05.

Table 1 and Figure 1 indicate that the dynamics of flavonoid components during the flowering and fruiting stages differ markedly between *Rosa xanthina* f. *normalis* (single-flowered) and *Rosa xanthina* ‘*Plena*’ (double-flowered). In the single-flowered form, total flavonoid content during the flowering stage was significantly higher than that in the double-flowered form, and the full-bloom stage represented a critical period for total flavonoid accumulation; its content then declined significantly after the onset of the fruiting stage. In the double-flowered form, no significant differences in total flavonoid content were observed among the developmental stages during the flowering period.

In terms of rutin content, both forms presented a V-shaped variation trend during flowering, with the order: bud stage > senescence stage > full-bloom stage. Overall, rutin content was higher in the single-flowered form across all stages, and it continuously declined with fruit maturation during the fruiting period.

The accumulation patterns of hyperoside and quercetin differed significantly between the two forms. For *Rosa xanthina* f. *normalis*, both compounds accumulated at high levels in the bud stage and gradually decreased with floral development, whereas their maximum accumulation in *Rosa xanthina* f. ‘*Plena’* occurred at the senescence stage.

Anthocyanins content showed a consistent variation trend in both forms: it accumulated continuously with flower opening during flowering and peaked at the senescence stage. In *Rosa xanthina* f. *normalis*, anthocyanins content increased sharply during the fruiting period and reached its maximum at the mature stage, which was considerably higher than that at the young fruit and color-change stages.

### 2.2. Correlation Analysis of Endogenous Flavonoids in Rosa xanthina f. normalis and Rosa xanthina ‘Plena’ Across Different Developmental Stages

**Figure 2:**
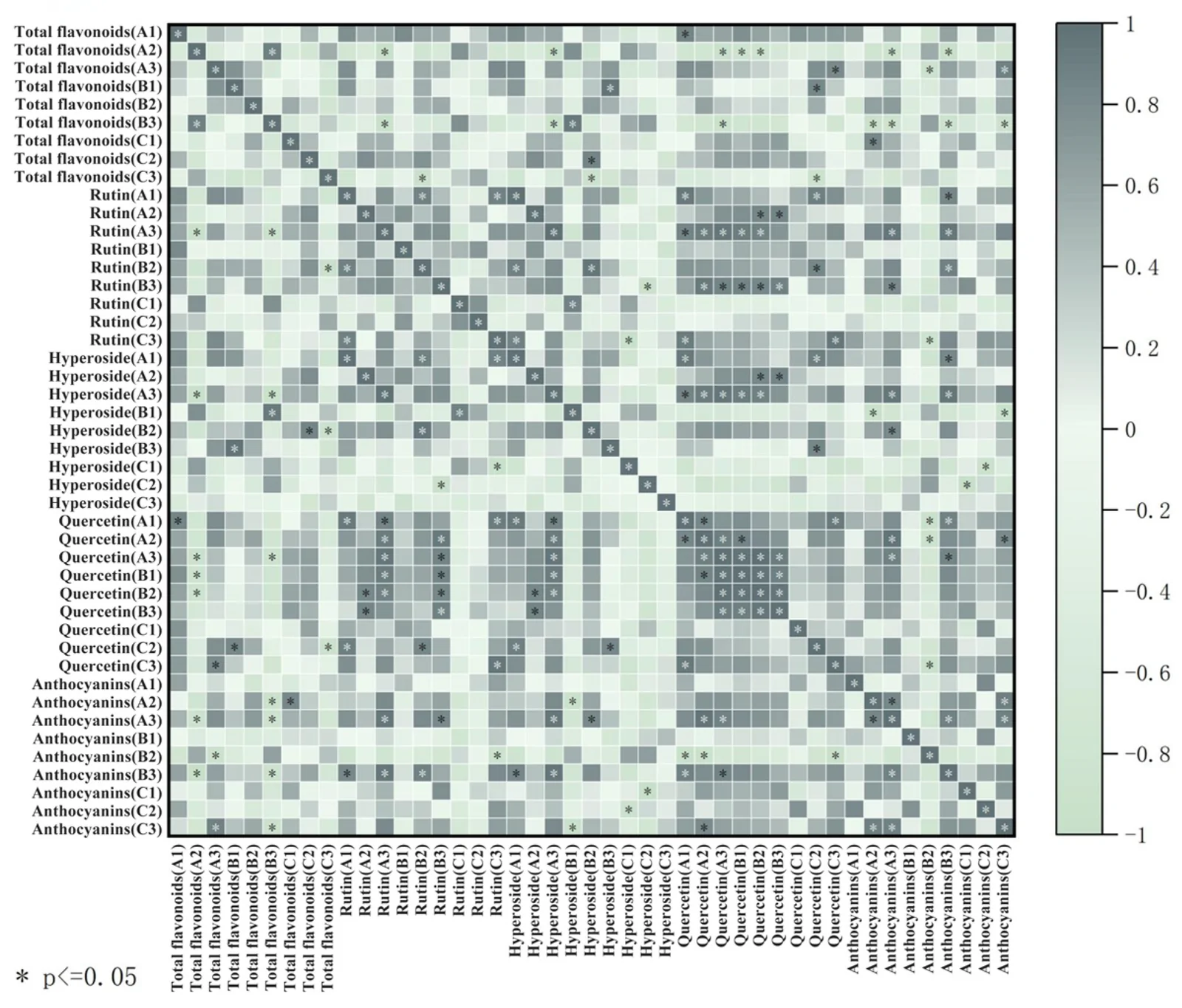
Correlation heatmap of flavonoids in single- and double-flowered *Rosa xanthina*. Significant correlations are marked with asterisks (*p* ≤ 0.05).

As shown by the results of the correlation analysis (Figure 2), the active components of *Rosa xanthina* can be divided into two major stable clusters across the sampled flower- and fruit-development stages. This clustering pattern represents an intrinsic species-specific property and remains unaffected by petal type or growth stage.

#### 2.2.1. Flavonoid Correlation Clusters

**Figure 3:**
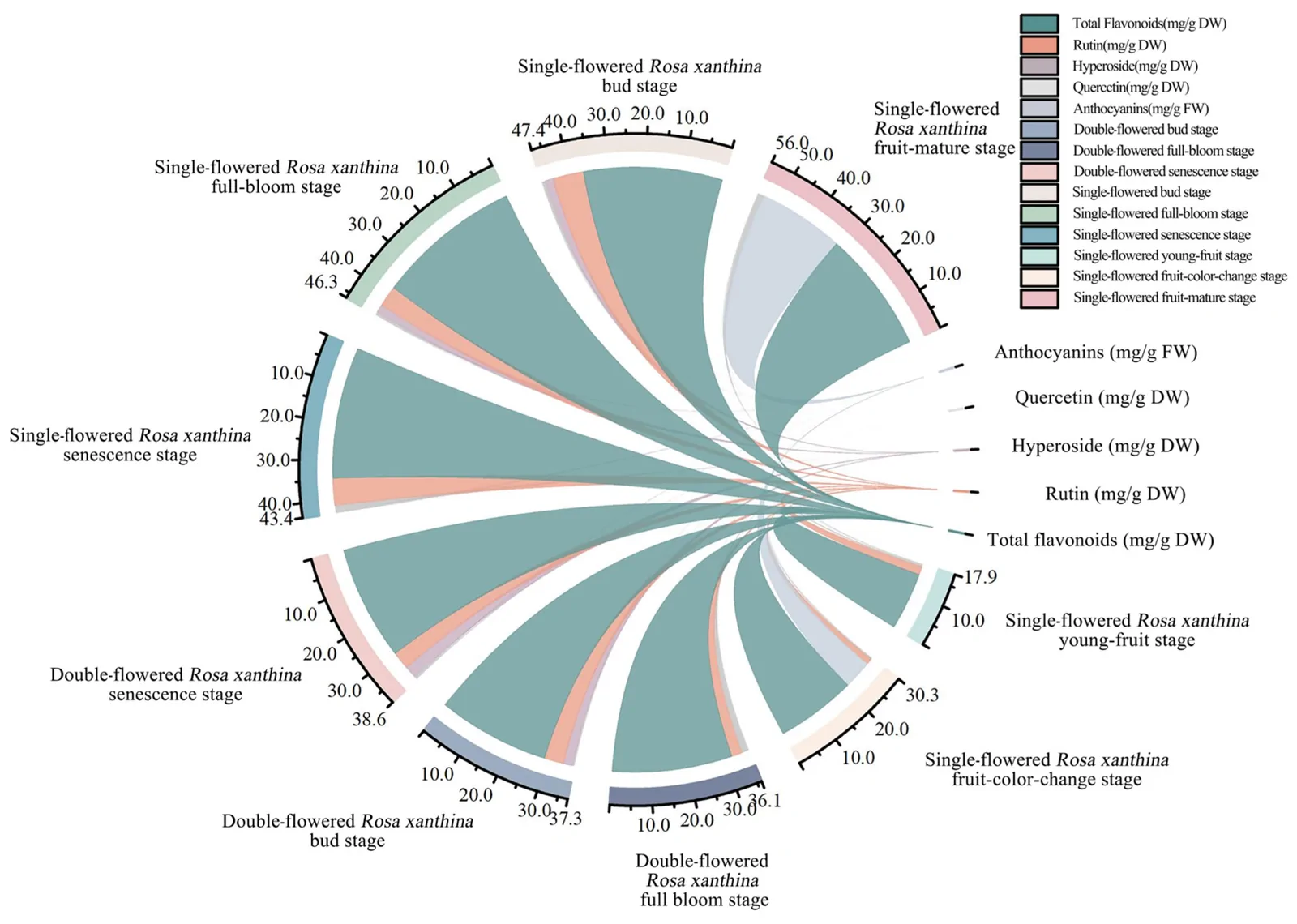
Proportional composition of flavonoids in single- and double-flowered *Rosa xanthina* across different developmental stages.

A strong positive correlation was found among total flavonoids, rutin, and hyperoside (*r* ≥ 0.817, *p* < 0.001), representing the most stable positive correlation combination across all samples (Figure 2). Together, these three components accounted for more than 35% of the total flavonoid content (Figure 3). Their positive association was stronger in single-flower flowers than in double-flower flowers and stronger during the flowering stage than during the fruiting stage (Figure 2). At the bud stage of single-flower flowers, the contents of total flavonoids and rutin reached the highest levels among all samples, and their correlation coefficient exceeded that observed in double-flower flowers at the same stage. During the wilting stage of double-flower flowers, the hyperoside content peaked, and its correlation with total flavonoids was stronger than that in single-flower flowers at the equivalent stage. At the fruiting stage, the metabolic center shifted toward anthocyanins, and the positive associative relationships among flavonoids weakened significantly. Petal type and growth stage influenced the strength of these associative relationships but did not alter their direction.

Quercetin showed a moderate positive correlation with total flavonoids and rutin, and a weak positive correlation with hyperoside, maintaining a positive association overall, which suggests the existence of a shared regulatory mechanism in their biosynthetic pathways. Only at the single-flower full-bloom stage, due to stage-specific metabolic regulation, quercetin and total flavonoids diverged slightly, causing a transient fluctuation in their coordination; nevertheless, the stability of the metabolic network across the entire flowering period remained higher than that during the fruiting period.

#### 2.2.2. Anthocyanins-Flavonoid Correlation Cluster

Anthocyanins exhibited strong negative correlations with rutin (*r* = −0.845) and total flavonoids (*r =* −0.803) (*p* < 0.001), representing the most pronounced negative-association combination in the whole sample (Figure 2). The antagonism was stronger in single-flower than in double-flower flowers and stronger at the fruit stage than at the flowering stage. The mean anthocyanins content at each stage of single-flower flowers was significantly higher than that of double-flower flowers, and the anthocyanins–rutin correlation coefficient was also higher in single-flower flowers than in double-flower flowers at the same stage. The negative-association effect in single-flower flowers was significantly stronger at the fruit stage (especially at maturity) than at the flowering stage, whereas the weakest antagonism occurred in double-flower flowers at the flowering stage. At the young fruit stage, when anthocyanins content was extremely low, no significant negative-association was detected; as anthocyanins accumulated with fruit ripening, the negative-association intensity gradually increased. Petal type and growth stage affected only the strength of the antagonism, not its direction.

Anthocyanins showed a moderate negative correlation with hyperoside (*r* = −0.758, *p* < 0.05), suggesting that quercetin metabolism is relatively independent of the anthocyanins pathway. Only during the ripening stage of single-flowered flowers, when anthocyanins content peaked, did hyperoside decline less than rutin, leading to a stage-specific difference in negative-association intensity. However, this did not alter the core pattern that higher anthocyanins content is associated with stronger negative-association, and the stability of the negative-association network during the fruiting stage remained higher than that during the flowering stage.

### 2.3. Comprehensive Advantage Categories of Flavonoids in Rosa xanthina f. normalis and Rosa xanthina ‘Plena’ at Different Growth Stages

**Figure 4:**
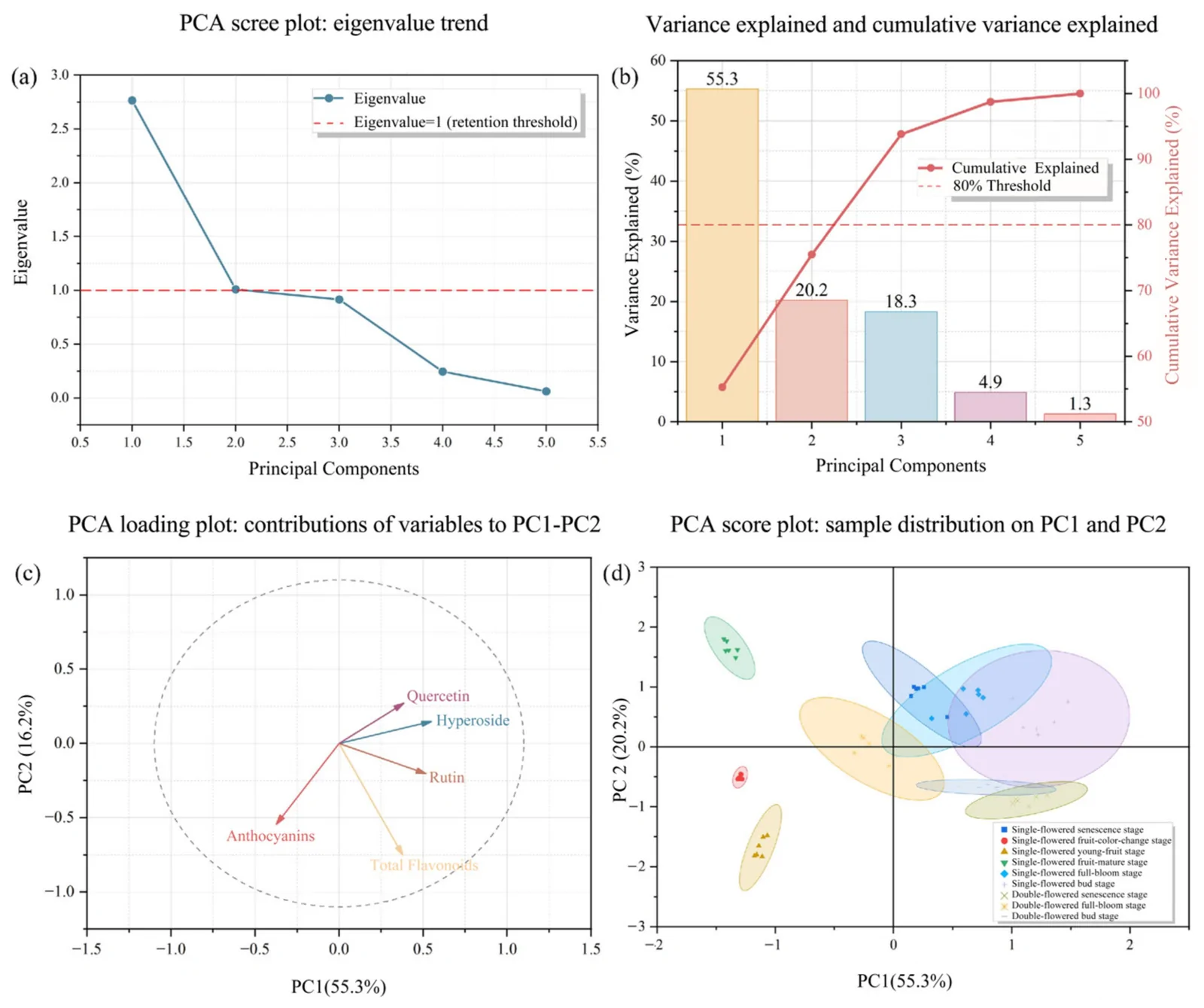
PCA analysis results of five flavonoids in *Rosa xanthina*. (**a**) PCA scree plot: eigenvalue trend; (**b**) Variance explained and cumulative variance explained; (**c**) PCA loading plot: contributions of variables to PC1-PC2; (**d**) PCA score plot: sample distribution on PC1 and PC2.

Principal component analysis (PCA) results (Figure 4) show that the first three PCs together account for 93.80% of the cumulative variance, effectively capturing all the information contained in the original five indicators. PC1 explains 55.28% of the variance and serves as the core axis characterizing growth stages, clearly separating flowering and fruiting samples: hyperoside and rutin exhibit high positive loadings, mainly corresponding to samples with high contents during the flowering period, whereas anthocyanins show high negative loadings, mainly associated with samples rich in anthocyanins during the fruiting period. PC2 accounts for 20.20% of the variance and acts as a trade-off axis for total flavonoids, distinguishing samples with high versus low total flavonoid levels: total flavonoids and anthocyanins have high negative loadings, corresponding to single-flowered full-bloom samples with high total flavonoids and fruiting samples with high anthocyanins, while the positive direction primarily characterizes single-flowered young fruit samples with low total flavonoid contents. PC3 had a variance contribution rate of 18.32% and represented the quercetin–anthocyanins synergy axis. This axis distinguished samples with high levels of both quercetin and anthocyanins from those with low levels of both: the positive direction exhibited high loadings for both compounds, corresponding to the double-flower senescence stage and the fruit stage, whereas the negative direction primarily characterized samples from the single-flower senescence stage, in which both compounds were relatively low. The sample clustering results indicated that samples from different developmental stages can be grouped into three clusters: the flowering-stage high-PC1 cluster (suitable for extracting rutin and hyperoside), the single-flower young fruit low-component cluster (with no extraction value), and the fruit-stage low-PC1 low-PC2 cluster (suitable for extracting anthocyanins).

## 3. Discussion

### 3.1. Changes in Flavonoid Compounds of Rosa xanthina f. normalis and Rosa xanthina ‘Plena’ at Different Stages

This study demonstrates that significant differences exist in the total flavonoid, flavonoid monomer, and anthocyanins contents of single-flowered and double-flowered *Rosa xanthina* at different developmental stages, indicating that the accumulation of its secondary metabolites exhibits distinct germplasm differences and developmental rhythmicity.

The total flavonoid content was significantly higher in single-flowered flowers than in double-flowered ones. This finding is consistent with previous reports that single-flowered white roses have higher flavonoid levels than double-flowered stocks [17], and supports the view that an increase in petal number and well-developed basal tissues may lead to a relative decrease in flavonoid accumulation. In both types of *Rosa xanthina*, total flavonoid content peaked during the flowering period (full bloom) and decreased significantly during the fruiting period. This trend aligns with findings by Yanlin An et al. in closely related species, suggesting that this pattern is somewhat conserved among Rosaceae plants [18].

The accumulation patterns of individual flavonoids exhibit distinct divergence. Rutin levels peak during the bud stage in both single-flowered and double-flowered *Rosa xanthina* and decrease significantly during the fruiting stage. This trend resembles the accumulation pattern of rutin in jasmine [19] and aligns with the regulatory mechanism wherein nutrient allocation shifts toward fruit development and the rutin biosynthetic pathway is downregulated after plants enter the fruiting stage [20]. The content of hyperoside is high during the flowering stage and low during the fruiting stage; specifically, it peaks at the senescence stage in double-flowered *Rosa xanthina* This finding is consistent with the conclusion that hyperoside primarily accumulates during the flowering stage in Hypericum perforatum [21], suggesting that its synthesis is closely associated with petal development. Quercetin content is low in both types of *Rosa xanthina* and is not a core flavonoid component; however, its level during the flowering stage is significantly higher in double-flowered than in single-flowered flowers, with a pronounced difference particularly at the senescence stage. This phenomenon is similar to research results reported for double-flowered ground cover chrysanthemum (Chrysanthemum × morifolium ‘Ground Cover’) [22], indicating that the double-flower trait may influence the regulatory mechanisms of quercetin synthesis.

The accumulation pattern of anthocyanins is opposite to that of flavonoids; the content is low during the flowering stage and increases substantially with the ripening process during the fruiting stage. This is consistent with the characteristic of massive anthocyanin accumulation in the late stages of fruit ripening in species such as peach (*Prunus persica*) and Trachycarpus fortunei [23,24], serving as a typical metabolic marker of fruit ripening. The limited synthesis of anthocyanins during the flowering stage may be attributed to the fact that genes associated with their synthesis are expressed only in the epidermal cells of petals [25].

In summary, the secondary metabolism of *Rosa xanthina* exhibits distinct developmental stage-specificity and petal-type differences. Flavonoids mainly accumulate during the flowering period, whereas anthocyanins reach their peak synthesis during the fruiting period. These findings provide a basis for elucidating the metabolic regulation mechanisms of secondary metabolites in *Rosa xanthina* and determining the appropriate harvesting time.

### 3.2. Intrinsic Associations of Flavonoids in Rosa xanthina f. normalis and Rosa xanthina ‘Plena’ at Different Stages

The “synergy-antagonism” metabolic correlation among the components is also an important finding of this study. Correlation analysis identified two major metabolic clusters, integrating the dynamic accumulation patterns of active constituents in *Rosa xanthina* from a systems perspective. Total flavonoids exhibited significant strong positive correlations with rutin and hyperoside (*r* = 0.82, *p* < 0.001), suggesting that these three compounds share phenylalanine as a metabolic precursor. Research by Kong J indicates that phenylalanine enters the phenylpropanoid pathway via PAL, serving as the common starting point for flavonoid biosynthesis [26]. In contrast, anthocyanins and flavonols showed a significant negative correlation (*r* = −0.67, *p* < 0.01), reflecting competitive relationships for metabolic flux between branching pathways. Genetic experiments by Yao-Wu Y et al. demonstrated that restoring FLS gene expression locally inhibited anthocyanin accumulation in petals, indicating direct competition between flavonols and anthocyanins for the key substrate dihydroflavonol [27]. This “positive-negative associative” metabolic pattern exhibits distinct variations across developmental stages. During the flowering stage, metabolic resources are preferentially allocated to the co-ordinated accumulation of flavonoids to meet the physiological demands of floral organs for antioxidant activity, biological defense, and pollinator attraction [28]. Upon entering the fruiting stage, the metabolic focus shifts significantly; flavonoid levels generally decline, whereas anthocyanins become the primary accumulated products. This pattern is highly consistent with observations in the fruit development of species such as bog bilberry (*Vaccinium uliginosum* L.), where flavonoids peak early and then decrease, while anthocyanins accumulate substantially during late maturation [29]. These findings suggest that this metabolic reallocation strategy may be closely associated with adaptive functions during fruit ripening, including pigment deposition, enhanced stress resistance, and seed dispersal.

In summary, the accumulation of flavonoids in *Rosa xanthina* is regulated not only by stock and developmental stage but also profoundly influenced by the positive-negative associative and competitive relationships among metabolic pathways. This finding provides a new perspective for understanding the accumulation patterns of plant secondary metabolites from a systemic metabolic standpoint.

### 3.3. Dominant Categories of Flavonoids in Rosa xanthina f. normalis and Rosa xanthina ‘Plena’ at Different Stages

By applying dimensionality reduction, the PCA condensed the complex interrelationships among five classes of active constituents into three core dimensions. This yielded an evaluation framework for characterizing differences in *Rosa xanthina* active constituents: the framework is structured primarily by developmental stage differentiation, secondarily by constituent concentration, and supplemented by specialized constituents. The results established the following regulatory priority in active constituent metabolism: developmental stage (PC1, 55.28%) is the foremost regulatory factor, followed by the concentration of core constituents (PC2, 20.20%), with the accumulation of specialized constituents (PC3, 18.32%) last. From an applied perspective, the PCA-derived screening model combining principal component loadings with cluster groupings enables rapid determination of sample class and potential development direction based solely on the concentrations of key constituents such as rutin and anthocyanins. This obviates the need for full-component profiling, thereby effectively reducing detection costs and shortening screening cycles.

Based on the above results, this study proposes targeted application strategies. In terms of variety selection and breeding, priority should be given to single-flowered *Rosa xanthina* f. *normalis*, enhancing the high accumulation traits of anthocyanins at the fruit ripening stage and rutin at the flower bud stage. The double-flowered type should be selectively bred into strains with high hyperoside accumulation at the senescence stage for specialized extraction. Hybridization can also integrate the high-anthocyanins trait of the single-flowered type and the high-hyperoside trait of the double-flowered type to develop varieties with multiple beneficial components. For harvest timing control, total flavonoid extraction achieved optimal results from the full-bloom stage (37.89 mg/g) of the single-flowered type, followed by the flower bud stage (36.28 mg/g). For rutin extraction, samples should be selected from the flower bud stage of the single-flowered type (8.03 mg/g). Hyperoside extraction should primarily focus on the senescence stage of the double-flowered type (3.19 mg/g), with the flower bud stage (2.41 mg/g) as a secondary source; the fruit stage consistently had levels below 0.5 mg/g, holding no value for extraction. Quercetin extraction is best suited to the senescence stage of the double-flowered type (0.32 mg/g); owing to its low overall content, combination with the extraction of other components is advisable to reduce costs. For anthocyanin extraction, harvesting should only target the fruit ripening stage of the single-flowered type (22.55 mg/g), which enables optimal extraction efficiency. In terms of resource allocation, *Rosa xanthina* ‘*Plena*’ can be primarily used for ornamental purposes, with the additional benefit of hyperoside extraction during the senescence stage. *Rosa xanthina* f. *normalis* can also serve ornamental functions while simultaneously enabling the development of rutin from the flower bud stage and anthocyanins from the fruit stage, thereby enhancing overall economic returns.

## 4. Materials and Methods

### 4.1. Overview of the Experimental Site

The trial sampling site is located in Taigu District, Jinzhong City, Shanxi Province. The area has a warm-temperate continental semi-arid monsoon climate, with four distinct seasons, abundant sunlight, dry conditions, and low precipitation. The mean annual temperature is approximately 10 °C, and annual precipitation ranges from 458 to 466 mm, concentrated mainly in June through September. Annual evaporation reaches 1765.9 mm, annual sunshine duration is 2500 to 2600 h, and the frost-free period lasts 175 to 180 days. The prevailing winds throughout the year are from the southwest and northwest.

The single-flowered and double-flowered *Rosa xanthina* plants used in the experiment were uniformly planted in the botanical garden of Shanxi Agricultural University. Daily management practices, including irrigation, fertilization, pruning, and pest and disease control, were kept consistent. The plants exhibited uniform growth and good vitality, eliminating any potential interference from environmental or managerial differences on the experimental results.

### 4.2. Experimental Reagents and Instruments

All experimental data in this study were statistically analyzed using SPSS 26.0 (IBM Corporation, Armonk, NY, USA). Origin 2025 (OriginLab Corporation, Northampton, MA, USA) was used for data plotting, curve fitting, and figure visualization. Principal component analysis and metabolite correlation analysis were performed using MetaboAnalyst 5.0.

### 4.3. Experimental Design

**Figure 5:**
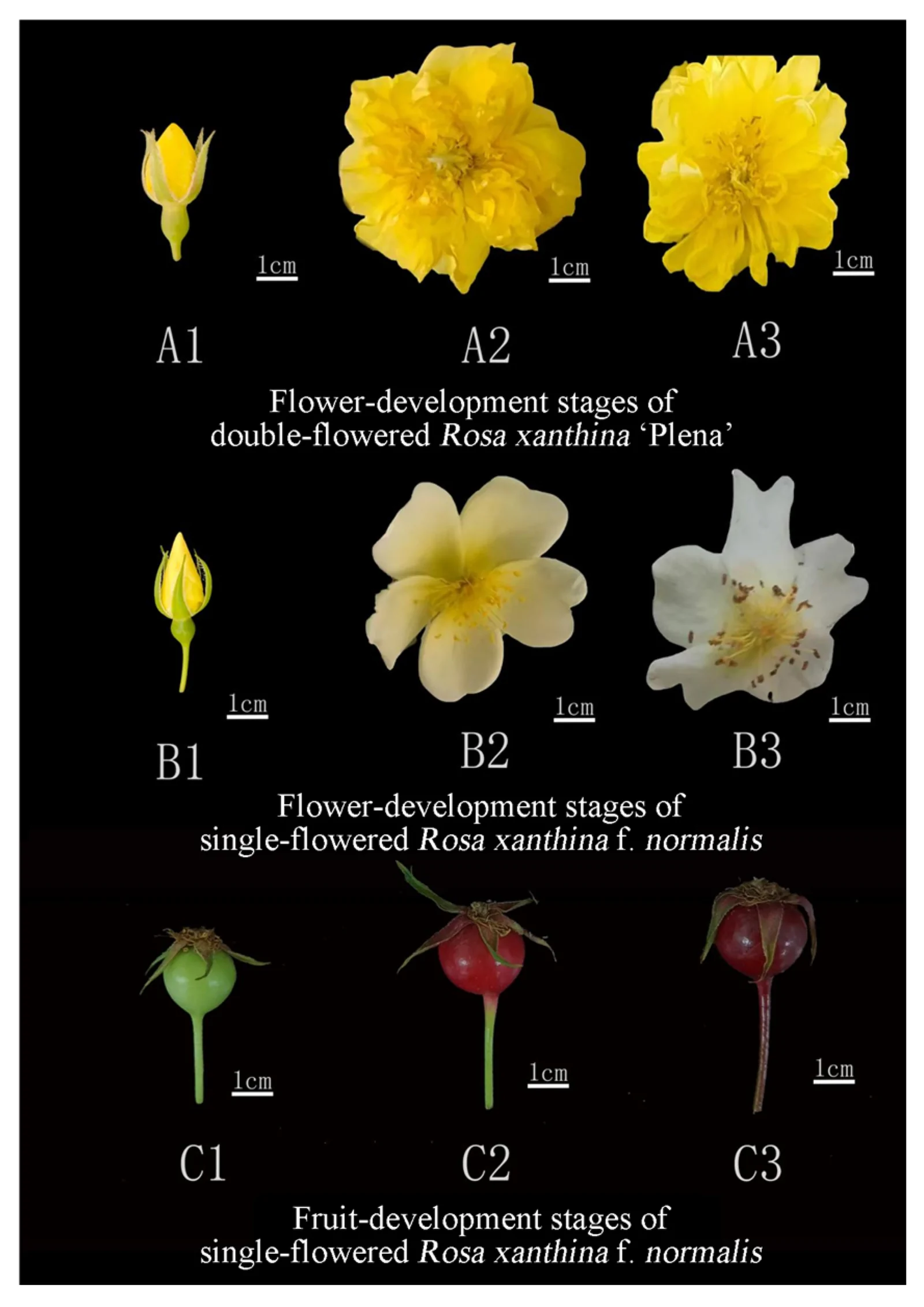
Phenotypic performance of *Rosa xanthina* at different developmental stages.

Based on the phenological development patterns of two *Rosa xanthina* forms, distinct flower- and fruit-developmental stages were selected for sampling (Figure 5). For the double-flowered form, the stages were bud stage (A1), full-bloom stage (A2), and senescence stage (A3). For the single-flowered form (which bears fruit normally), the stages were bud stage (B1), full-bloom stage (B2), and senescence stage (B3); additionally, three fruit developmental stages were supplemented: young fruit stage (C1), color-change stage (C2), and maturity stage (C3).

For each sampling period, six biological replicates were established to ensure the stability and representativeness of the experimental data. Each replicate was a mixed tissue sample collected from multiple healthy and disease-free plants. To minimize individual differences, sampling was consistently performed on the same batch of plants throughout the entire phenological period. Whole flowers and fruits were collected according to corresponding developmental stages. All samples were harvested from April to August 2024 strictly based on field phenological changes. After collection, each mixed sample was divided into two parts: one was immediately frozen in liquid nitrogen and stored at −80 °C, and the other was freeze-dried using a Scientz-18N freeze-dryer (Ningbo Scientz Biotechnology Co., Ltd., Ningbo, China), ground, and sieved through an 80-mesh sieve for subsequent metabolite determination. All extracts were detected in three technical replicates. The total flavonoid content was determined using the sodium nitrite–aluminum nitrate colorimetric method [30]. Three monomeric flavonoids, namely rutin, hyperoside, and quercetin, were quantified by high-performance liquid chromatography (HPLC) [15]. The anthocyanins content was measured by solvent-extraction spectrophotometry [31].

#### 4.3.1. Total Flavonoids Determination

Total flavonoids were determined via the sodium nitrite–aluminum nitrate colorimetric method. Briefly, 0.1 g sample powder was extracted with 8 mL of 75% ethanol under ultrasonic treatment (50 °C, 40 Hz, 60 min). After centrifugation at 5000 r·min^−1^ for 10 min, the supernatant was processed with 5% sodium nitrite and 10% aluminum nitrate, stood for 6 min sequentially, and reacted with 4% sodium hydroxide. After 15 min of color development, the absorbance was measured at 510 nm using a UV-2500 spectrophotometer (Techcomp, Shanghai, China). Rutin standard (≥98%, Solarbio, Beijing, China, batch No. 2451226002) was used for standard curve calibration. All other chemicals and reagents (sodium nitrite, aluminum nitrate, sodium hydroxide, and ethanol) were of analytical grade and were purchased from Sinopharm Chemical Reagent Co., Ltd. (Shanghai, China).

#### 4.3.2. HPLC Analysis of Rutin, Hyperoside and Quercetin

Rutin, hyperoside, and quercetin were quantified by HPLC (Thermo U3000, Thermo Fisher Scientific, Waltham, MA, USA). A total of 1.0 g sample powder was reflux-extracted with 10 mL of 70% ethanol for 1 h. The filtrate was passed through a 0.22 μm organic membrane before detection. Determination was conducted using an Athena-C18 column (250 mm × 4.6 mm, 5 μm; ANPEL Laboratory Technologies (Shanghai) Inc., Shanghai, China)at 25 °C, with a flow rate of 1.0 mL/min, injection volume of 20 μL, and detection wavelength of 360 nm. The mobile phase consisted of acetonitrile (A) and 0.1% phosphoric acid aqueous solution (B) with a gradient elution program. Acetonitrile and phosphoric acid were of chromatographic grade and were purchased from Sinopharm Chemical Reagent Co., Ltd. (Shanghai, China). Mixed standard solutions (≥98%, Solarbio, Beijing, China) were used for qualitative and quantitative analysis. Method validation including precision, stability, repeatability, and recovery was performed, while LOD and LOQ were not determined in this study.

#### 4.3.3. Anthocyanins Measurement

Anthocyanins were extracted using 0.1 mol/L HCl solution. In brief, 1 g fresh tissue was immersed in 10 mL HCl solution and incubated at 32 °C for 4 h. The absorbance was detected at 530 nm, and the results were expressed as relative anthocyanins content (OD_530_ ×10 per g DW). No commercial anthocyanins standard was applied in this experiment.

#### 4.3.4. Data Analysis

All data were expressed as the mean ± standard deviation (SD), and significant differences were determined at *p* < 0.05. This study employed one-way analysis of variance (one-way ANOVA) to calculate SST, SSA and SSE via SPSS statistical software, obtaining F-values and *p*-values. Pearson correlation analysis was applied to evaluate correlation strength using dual indicators of correlation coefficient (*r*) and significance (*p*). Principal component analysis (PCA) was used to identify comprehensive superior categories by analyzing the contribution rates of principal components and the loadings of each index. Graphs were plotted with Origin 2025.

## 5. Conclusions

This study used ANOVA, Pearson correlation analysis and PCA to characterize flavonoid accumulation patterns in single- and double-flowered Rosa xanthina. Flower-type difference was a key factor affecting metabolite levels. The single-flowered form had higher total flavonoids at the full-bloom stage, higher rutin at the bud stage, and higher anthocyanins at fruit-ripening stage, whereas the double-flowered genotype accumulated more hyperoside and quercetin at the senescence stage. Developmental stages exerted dynamic influences on flavonoid accumulation: total flavonoids peaked at full-bloom stage, rutin peaked at bud stage; hyperoside and quercetin reached maximum levels during senescence; obvious anthocyanin accumulation occurred in fruits of single-flowered plants.

Statistical associations existed among metabolites: total flavonoids showed strong positive correlations with rutin and hyperoside (*r* = 0.82, *p* < 0.001), while anthocyanins were significantly negatively correlated with flavonols (*r* = −0.67, *p* < 0.01). It should be noted that these correlations only reflect statistical relationships rather than causal or metabolic interactions.

PCA results reflected comprehensive differences in metabolite profiles across samples. The present work documents how phenological stages shape flavonoid accumulation in flowers and fruits of *Rosa xanthina*. Practically, these stage-dependent patterns indicate that harvest timing is at least as important as floral form in determining flavonoid yield. These variable accumulation characteristics of flavonoids and anthocyanins also provide basic reference for exploring the antioxidant potential of *Rosa xanthina*-derived metabolites. Since sampling was performed only at one site within a single growing season, multi-site and multi-year investigations on different natural populations are required to validate our findings.

## Figures and Tables

**Table 1:** Analysis of variance for flavonoid compounds in different flowering and fruit-development stages of *Rosa xanthina*.

	Double-Flowered *Rosa xanthina* ‘*Plena*’			Single-Flowered *Rosa xanthina* f. *normalis*					
	Bud Stage (*n* = 6)	Full-Bloom Stage (*n* = 6)	Senescence Stage (*n* = 6)	Bud Stage (*n* = 6)	Full-Bloom Stage (*n* = 6)	Senescence Stage (*n* = 6)	Young- Fruit Stage (*n* = 6)	Fruit-Color-Chang Stage (*n* = 6)	Fruit-Mature Stage (*n* = 6)
Total flavonoids (mg/g DW)	29.33 ± 0.48 b	31.33 ± 0.61 a	29.93 ± 0.91 b	36.28 ± 2.25 cd	37.89 ± 2.15 c	34.34 ± 0.96 e	14.96 ± 1.38 h	20.92 ± 0.31 g	31.78 ± 0.38 f
Rutin (mg/g DW)	5.06 ± 0.31 a	2.79 ± 0.16 c	4.64 ± 0.16 b	8.03 ± 0.33 d	5.45 ± 0.18 f	7.05 ± 0.18 e	2.13 ± 0.04 g	1.50 ± 0.04 h	1.22 ± 0.15 i
Hyperioside (mg/g DW)	2.41 ± 0.08 b	1.47 ± 0.33 c	3.19 ± 0.44 a	2.12 ± 0.45 d	2.08 ± 0.19 d	1.17 ± 0.02 e	0.45 ± 0.02 f	0.41 ± 0.01 g	0.30 ± 0.01 h
Quercetin (mg/g) DW	0.27 ± 0.03 a	0.09 ± 0.07 b	0.32 ± 0.04 a	0.24 ± 0.03 c	0.11 ± 0.01 d	0.04 ± 0.05 e	0.09 ± 0.01 g	0.08 ± 0.00 h	0.13 ± 0.01 f
Anthocyanins (mg/g FW)	0.28 ± 0.05 b	0.38 ± 0.10 b	0.51 ± 0.10 a	0.73 ± 0.07 c	0.78 ± 0.12 c	0.83 ± 0.09 c	0.24 ± 0.10 f	7.43 ± 0.24 e	22.55 ± 1.87 d

Note: Identical letters indicate no significant difference between groups; different letters indicate a significant difference.

## Data Availability

The data presented in this study are available on request from the corresponding author.
